# Electroacupuncture-inspired neuroimmune modulation in sepsis: evidence appraisal and ICU trial-design priorities

**DOI:** 10.3389/fmed.2026.1891245

**Published:** 2026-07-30

**Authors:** Youjuan Qian, Chenghao Lin, Hui Lin, Yingxue Leng, Kai Xie, Yue Shan, Lili Xu

**Affiliations:** 1 Department of Anesthesiology, Shaoxing People's Hospital, The First Hospital of Shaoxing University, Shaoxing, China; 2 School of Medicine, Shaoxing University, Shaoxing, Zhejiang, China; 3 Department of Medical Research Center, Shaoxing People's Hospital, Zhejiang University Shaoxing Hospital, Shaoxing, Zhejiang, China

**Keywords:** electroacupuncture, immunophenotyping, inflammatory reflex, neuroimmune modulation, sepsis

## Abstract

Electroacupuncture (EA) has emerged as a biologically plausible adjunctive strategy for modulating neuroimmune dysregulation in sepsis. However, several recent reviews have already summarized anti-inflammatory mechanisms, ST36-centered animal evidence, organ-protective effects, and pooled clinical signals of acupuncture or EA in experimental and clinical sepsis. The unmet need is therefore not another catalog of positive pathways, but a critical translational framework that asks when, in whom, and how EA-inspired neuromodulation should be evaluated within the clinical complexity of septic intensive care. This review integrates mechanistic, preclinical, clinical, safety, and trial-design literature to appraise the evidence hierarchy, clarify sepsis immunophenotype-based patient-selection logic, and identify ICU-specific barriers to clinical translation. We emphasize somato-autonomic reflexes, inflammatory reflex signaling, vagal-adrenal and cholinergic anti-inflammatory pathways, macrophage and T-cell regulation, gut barrier immunity, immune suppression, and biomarker-guided stratification. Current clinical evidence suggests possible adjunctive signals, particularly in sepsis-associated gastrointestinal dysfunction and inflammatory biomarker modulation, but available trials are small, heterogeneous, frequently unblinded, and often use usual-care rather than sham comparators. Accordingly, reported mortality signals should be interpreted as hypothesis-generating rather than established clinical efficacy. We propose that future research should prioritize phenotype-specific, sham-controlled, multicenter trials with standardized stimulation protocols, organ-specific endpoints, immune and barrier biomarkers, and rigorous ICU safety governance, with gastrointestinal dysfunction serving as the most actionable initial indication. Closed-loop or artificial intelligence-guided EA should be regarded only as a future, physician-supervised engineering perspective. EA remains a biologically plausible but clinically unproven adjunctive neuromodulatory strategy in sepsis; its value for hard outcomes such as mortality, durable organ protection, and long-term recovery requires confirmation in rigorously designed ICU trials.

## 1 Introduction

Sepsis is no longer conceptualized as a simple systemic inflammatory response. The Sepsis-3 definition emphasizes life-threatening organ dysfunction caused by a dysregulated host response to infection (1). Global estimates indicate that sepsis contributed to approximately 48.9 million cases and 11.0 million sepsis-related deaths in 2017, corresponding to nearly one fifth of all global deaths (2). Contemporary international guidelines prioritize time-sensitive antimicrobial therapy, source control, hemodynamic optimization, vasopressor support, corticosteroids in selected shock phenotypes, mechanical ventilation, renal replacement therapy, nutrition, and other organ-support strategies (3). These measures remain the therapeutic foundation of sepsis care.

Nevertheless, sepsis-associated organ dysfunction frequently progresses despite technically adequate source control and hemodynamic support. One explanation is that sepsis is immunologically dynamic. Early inflammatory amplification, endothelial activation, complement and coagulation dysregulation, mitochondrial dysfunction, lymphocyte apoptosis, impaired antigen presentation, and macrophage exhaustion may overlap within the same patient (4, 5). This complexity has undermined many single-target anti-inflammatory and immunostimulatory strategies. A rational adjunctive therapy would therefore need to be titratable, context-sensitive, and less likely to abolish host antimicrobial defense.

**Figure 1:**
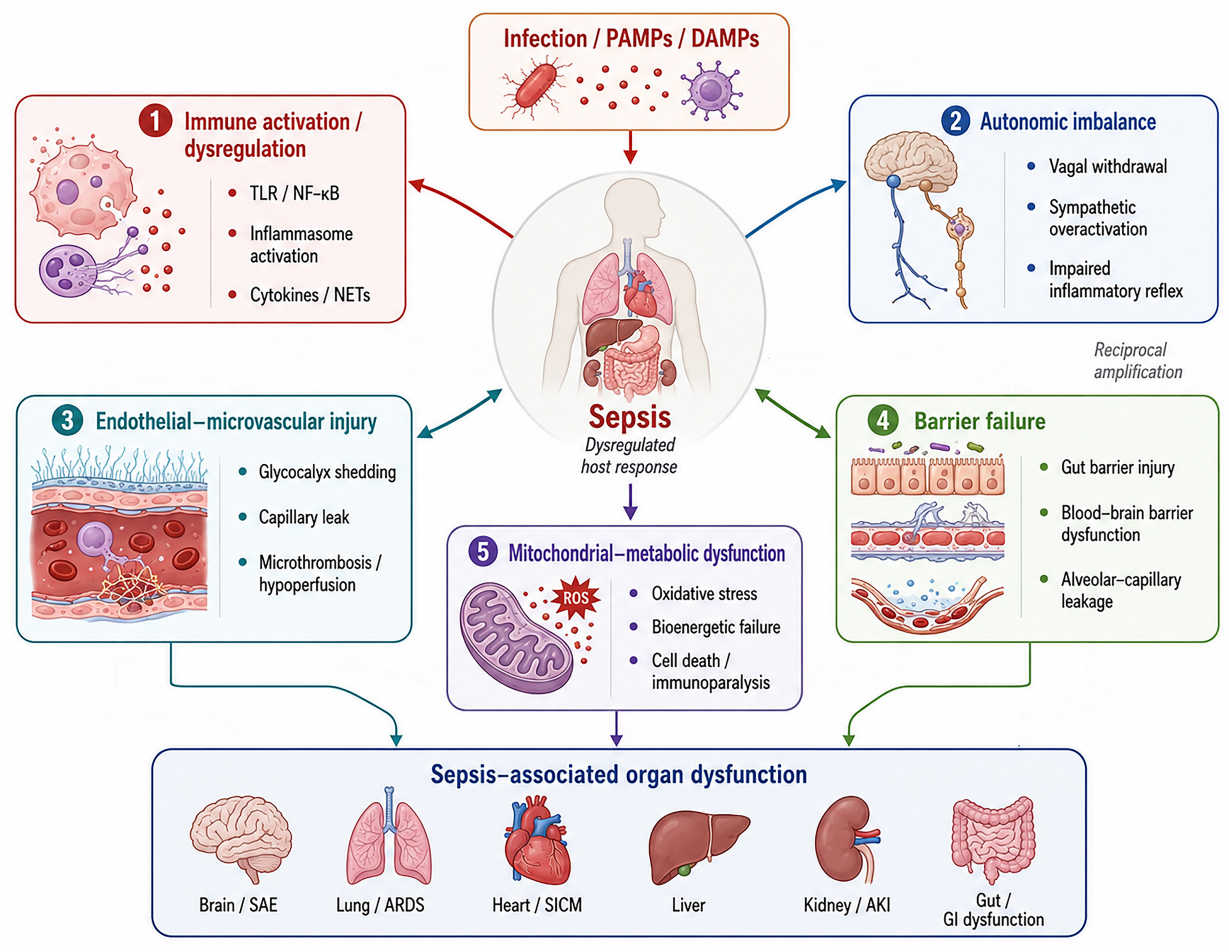
Neuroimmune dysregulation in sepsis-associated organ dysfunction. Sepsis-associated organ dysfunction arises from reciprocal interactions among innate immune activation, autonomic imbalance, endothelial-microvascular injury, epithelial barrier failure, mitochondrial-metabolic dysfunction, and organ-specific vulnerability. This framework provides the pathophysiological rationale for studying EA as an adjunctive neuroimmune intervention rather than as an anti-infective therapy.

Acupuncture has historically been used in inflammatory and gastrointestinal disorders. Bioelectronic medicine and neuroimmunology provide a mechanistic framework for understanding how peripheral neural stimulation may influence immune responses (6). The anatomical discovery of hardwired neuroimmune circuits, such as the vagal-splenic axis and the cholinergic anti-inflammatory reflex, demonstrates that the central nervous system continuously monitors and intricately calibrates peripheral innate immunity (7, 8). Within this framework, electroacupuncture (EA) can be viewed as a reproducible form of somatosensory electrical stimulation rather than as a conventional anti-infective therapy. By applying precise electrical parameters to specific peripheral somatosensory coordinates (9), EA may engage autonomic and neuroendocrine reflexes that modulate inflammatory responses in experimental settings. This allows for the precise, dose-dependent dampening of localized hyperinflammation and the preservation of critical organ barriers, circumventing the systemic toxicity of conventional pharmacological agents (10). Recent reviews have summarized the anti-inflammatory and organ-protective effects of EA in sepsis, including a systematic review of excessive inflammatory responses and organ damage (11) and a 2025 rodent-literature review focused on ST36-mediated neuro-endocrine-immune regulation, exosome biology, and multi-organ protection (12). A previous ST36-focused systematic review of experimental sepsis also highlighted substantial laboratory evidence but emphasized methodological heterogeneity and limited translatability (13). Therefore, the present review shifts the focus from whether EA is biologically plausible to how it should be critically evaluated, safely implemented, and rigorously tested as an adjunctive neuroimmune intervention in sepsis. We integrate selected concepts from newer mechanism-oriented literature, including local acupoint biology, afferent recruitment, gut microbiota-metabolite axes, and stimulation-dose standardization, while deliberately avoiding overextension from non-sepsis EA fields. The central argument is that EA can be considered only as an adjunct to guideline-based sepsis care and that its potential clinical value should be tested through ICU-compatible, phenotype-specific, sham-controlled trials with organ-specific endpoints and robust safety governance. As illustrated in Figure 1, sepsis-associated organ dysfunction arises from reciprocal interactions among innate immune activation, autonomic imbalance, endothelial-microvascular injury, barrier failure, and mitochondrial-metabolic dysfunction.

## 2 Scope, evidence audit, and distinction from previous reviews

This article is a critical translational review rather than a PRISMA-style systematic review; therefore, the search strategy was designed to support transparent evidence mapping rather than quantitative evidence synthesis. It uses previous systematic reviews, rodent-literature reviews, mechanistic studies, randomized trials, ICU safety evidence, clinical guidelines, and reporting standards to identify translational gaps and trial-design priorities. Because recent reviews have already cataloged ST36-centered animal evidence and broad anti-inflammatory mechanisms in sepsis (11, 12), the present review does not reproduce a complete inventory of all ST36 animal studies. Instead, it asks which mechanistic signals are sufficiently credible to justify clinical testing, which ICU factors may invalidate simple extrapolation, and which endpoints and safety criteria are needed for rigorous evaluation.

To improve transparency, a targeted literature search was performed in PubMed/MEDLINE, Embase, Web of Science, Cochrane Library, and ClinicalTrials.gov. The search covered studies published from database inception to May 2026. Search terms combined sepsis-related keywords (“sepsis,” “septic shock,” “endotoxemia,” “lipopolysaccharide,” “cecal ligation and puncture,” “sepsis-associated organ dysfunction”) with acupuncture-related keywords (“acupuncture,” “electroacupuncture,” “transcutaneous electrical acupoint stimulation,” “ST36,” “Zusanli,” “PC6,” “Neiguan,” “vagus nerve,” “cholinergic anti-inflammatory pathway,” “bioelectronic medicine”) and implementation-related keywords (“intensive care,” “ICU,” “safety,” “adverse events,” “contraindication,” “randomized controlled trial,” “sham acupuncture,” “trial design,” “heart rate variability,” “biosensor,” “artificial intelligence”). Eligible evidence included mechanistic animal studies, human randomized or controlled clinical studies, systematic reviews/meta-analyses, ICU safety literature, reporting guidelines, sepsis guidelines, and clinical trial protocols relevant to EA or acupuncture-based interventions in sepsis. Studies were prioritized when they directly addressed sepsis, endotoxemia, CLP, sepsis-associated organ dysfunction, ICU implementation, or clinical trial design. Non-sepsis acupuncture studies were used only when they clarified general biological or methodological principles, and were explicitly identified as indirect evidence. Retracted publications were not used as efficacy evidence. No quantitative synthesis or formal risk-of-bias scoring was performed because the aim was translational evidence mapping rather than meta-analysis.

The evidence was interpreted according to hierarchy and clinical relevance. Preclinical data were treated as mechanistic plausibility, not proof of ICU efficacy. Pooled effects from small clinical trials were interpreted as hypothesis-generating when GRADE certainty was low or very low. We also distinguished between statistical signals and clinically actionable outcomes. A reduction in inflammatory biomarkers or disease-severity scores may support biological activity, but it does not establish survival benefit, durable organ protection, or routine ICU readiness. Retraction status and methodological quality were also considered.

### 2.1 Immunological framework and patient-selection logic

A central premise of this review is that EA should not be considered a uniform anti-inflammatory intervention for all septic patients. Human sepsis is immunologically heterogeneous, with overlapping hyperinflammatory, immunosuppressed, endothelial-injury, metabolic, and mixed phenotypes (4, 5, 14–16). Patients with early hyperinflammation, preserved autonomic responsiveness, gastrointestinal barrier injury, and measurable inflammatory activation may theoretically differ from patients with profound lymphopenia, reduced monocyte HLA-DR, secondary infection, or immune exhaustion (5, 16). Therefore, future EA studies should incorporate immune phenotyping rather than enrolling unselected septic populations. Candidate stratification variables include lymphocyte count, monocyte HLA-DR, neutrophil-to-lymphocyte ratio, cytokine profiles, lactate kinetics, shock status, vasopressor exposure, organ-dysfunction trajectories, and autonomic signal quality when available (14–16). This immunophenotype-guided approach is essential to distinguish potentially modifiable neuroimmune activation from late-stage immunoparalysis or irreversible organ failure.

To clarify the novelty of the present review, Table 1 compares our scope with recent reviews of acupuncture or EA in sepsis. Previous reviews have primarily summarized ST36-centered animal evidence, anti-inflammatory mechanisms, organ-protective pathways, gastrointestinal dysfunction, or pooled clinical efficacy. By contrast, the present review focuses on evidence hierarchy, sepsis immune phenotypes, ICU-specific confounders, safety governance, and phenotype-specific trial design. Thus, its primary contribution is not the addition of another mechanistic catalog, but the development of a translational framework for determining when, in whom, and how EA-inspired neuroimmune modulation should be tested in septic ICU populations.

**Table 1:** Comparison between the present review and previous reviews on acupuncture/electroacupuncture in sepsis.

Review	Main focus	Evidence base	Key contribution	Limitation addressed by the present review
Lai et al. (13)	ST36 in experimental sepsis	Animal studies	Systematic summary of ST36 preclinical evidence	Limited discussion of ICU implementation, immunophenotyping, and trial design
Yang et al. (11)	Biological mechanisms of acupuncture in sepsis-related inflammation and organ injury	Mechanistic and preclinical studies	Broad catalog of anti-inflammatory and organ-protective mechanisms	Less emphasis on evidence hierarchy, ICU confounders, and clinical readiness
Zeng and Yan (12)	ST36-centered rodent literature and preventive-treatment concepts	Rodent studies	Focused discussion of neuro-endocrine-immune regulation and multi-organ protection	Mainly preclinical; limited focus on human sepsis heterogeneity and ICU trial feasibility
Xian et al. (71)	Clinical efficacy and safety of acupuncture as complementary therapy	RCT meta-analysis	Pooled estimates for disease severity, biomarkers, gastrointestinal outcomes, and mortality	Low/very-low certainty evidence; limited mechanistic and phenotype-specific trial-design appraisal
Present review	EA-inspired neuroimmune modulation in septic ICU care	Mechanistic, clinical, safety, implementation, and trial-design evidence	Evidence appraisal, sepsis immune phenotypes, ICU barriers, GI-focused trial framework, and safety governance	Addresses how, when, and in whom EA should be tested rather than simply listing mechanisms

To make this hierarchy explicit, Table 2 categorizes the available literature by study type, translational relevance, principal limitations, and overall certainty. This matrix is not intended to replace formal GRADE assessment, but to prevent mechanistic plausibility, preclinical efficacy, pooled low-certainty clinical signals, and speculative engineering concepts from being treated as equivalent levels of evidence.

**Table 2:** Evidence matrix and certainty assessment for electroacupuncture in sepsis.

Evidence category	Representative evidence	Main implication	Key limitations	Overall certainty
Local acupoint biology and afferent recruitment	Adenosine/A1 receptor signaling, fascia-associated mechanosensory activation, PROKR2Cre-marked sensory neurons at ST36	Supports the concept that EA acts through anatomically constrained somatosensory interfaces	Most data are from pain, endotoxemia, or non-ICU models; afferent recruitment in sedated, edematous, vasopressor-dependent patients is unproven	Moderate for biological mechanism; low for septic ICU translation
Somato-autonomic and inflammatory reflex mechanisms	Vagal-adrenal signaling, vagal-splenic cholinergic anti-inflammatory pathway, α7nAChR-dependent macrophage modulation	Provides a coherent neuroimmune rationale for adjunctive modulation of inflammation	Circuit behavior may differ with shock, exogenous catecholamines, lymphopenia, autonomic failure, and immune exhaustion	Moderate in preclinical models; low in human sepsis
Rodent sepsis and endotoxemia models	LPS and CLP models evaluating cytokines, survival, gut barrier, lung injury, brain injury, kidney injury, liver injury, and cardiac dysfunction	Suggests anti-inflammatory and organ-protective signals across multiple organs	Young healthy animals, pretreatment designs, limited supportive care, and model-specific immune kinetics limit generalizability	Low to moderate
Organ-specific clinical signals	Small RCTs and controlled studies assessing gastrointestinal dysfunction, intra-abdominal pressure, inflammatory biomarkers, APACHE II, immune indices, and selected organ outcomes	Suggests possible adjunctive activity, especially for gastrointestinal dysfunction	Small samples, single-center designs, limited blinding, usual-care comparators, variable EA protocols, and incomplete adverse-event reporting	Low
Systematic reviews and meta-analyses	Sepsis-focused acupuncture/EA meta-analyses reporting pooled effects on severity scores, biomarkers, intra-abdominal pressure, and 28-day mortality	Helps identify candidate outcomes and recurring signals	Pooled estimates are limited by low/very-low GRADE certainty, heterogeneity, possible publication bias, and frequent absence of sham controls	Low or very low
ICU safety and implementation evidence	Acupuncture safety literature, ICU implementation considerations, CONSORT-STRICTA reporting, contraindication guidance	Defines practical boundaries for protocol design and safety governance	Limited sepsis-specific adverse-event data; risks differ across coagulopathy, devices, edema, shock, and staffing environments	Low
AI-guided and closed-loop neuromodulation concepts	Bioelectronic medicine, HRV, biosensors, DECIDE-AI, TRIPOD+AI, CONSORT-AI	Offers a future roadmap for phenotype recognition, safety monitoring, and protocol adherence	No direct evidence for AI-guided or closed-loop EA in septic ICU populations; signal quality and regulatory validation remain unresolved	Very low/theoretical

## 3 Neuroimmune mechanisms under ICU-specific confounding

### 3.1 Local acupoint biology and afferent recruitment

Local acupoint biology provides an additional layer for understanding how peripheral stimulation may be converted into neuroimmune signals. Experimental work outside sepsis has shown that EA can increase local extracellular adenosine and activate adenosine A1 receptors, contributing to local antinociceptive effects (17). Other studies suggest that needling and electrical stimulation can interact with mechanosensitive somatosensory afferents and deep fascia. In sepsis-related neuroimmune research, the most influential finding is that low-intensity stimulation at the hindlimb ST36 region can recruit PROKR2Cre-marked sensory neurons innervating deep hindlimb fascia, thereby driving the vagal-adrenal anti-inflammatory axis in mice (9). These observations support a refined concept: the acupoint is not simply a symbolic location but a neuroanatomically and biophysically constrained interface. However, most local acupoint microenvironment studies derive from pain or non-sepsis models. Their relevance to mechanically ventilated, sedated, edematous, or vasopressor-dependent septic ICU patients remains indirect. Future studies should therefore measure afferent recruitment, autonomic output, local tissue impedance, edema, perfusion status, and stimulation tolerability rather than assuming that a stimulation protocol effective in rodents or ambulatory subjects recruits the same sensory circuits in critical illness.

### 3.2 Somato-autonomic reflexes and inflammatory reflexes

The best-established mechanistic rationale for EA in sepsis involves somato-autonomic reflexes and inflammatory reflex pathways. Sciatic nerve activation with EA was reported to control systemic inflammation in polymicrobial peritonitis through vagal activation of adrenal dopamine production (10). Vagus nerve stimulation attenuates systemic inflammatory responses to endotoxin, and the inflammatory reflex has been described as a neural circuit that senses and modulates peripheral inflammation (8, 18–20). In canonical models, vagal output influences splenic sympathetic signaling; norepinephrine from splenic nerve terminals acts on beta2-adrenergic receptors on choline acetyltransferase-positive T cells, which then release acetylcholine to activate alpha7 nicotinic acetylcholine receptors (α7nAChR) on macrophages (21). Activation of this pathway suppresses pro-inflammatory cytokine production, including TNF-α, without necessarily eliminating antimicrobial phagocytic capacity (22). This circuit provides a plausible basis for neuromodulation, but its translation to advanced septic shock is not guaranteed, particularly when lymphopenia, autonomic dysfunction, or macrophage exhaustion is present.

Across preclinical endotoxemia and CLP models, EA has been linked to several convergent inflammatory modules, including TLR4/MyD88/NF-κB signaling, HMGB1-related late inflammatory signaling, macrophage polarization, and programmed cell death pathways. However, these mechanisms are model-, organ-, acupoint-, and timing-dependent, and should not be interpreted as a uniform mechanism applicable to all septic patients (23–27). These mechanisms are biologically coherent but not equivalent to clinical efficacy. Many experiments use pretreatment before lipopolysaccharide (LPS) injection or cecal ligation and puncture, whereas ICU clinicians treat established sepsis (28).

**Figure 2:**
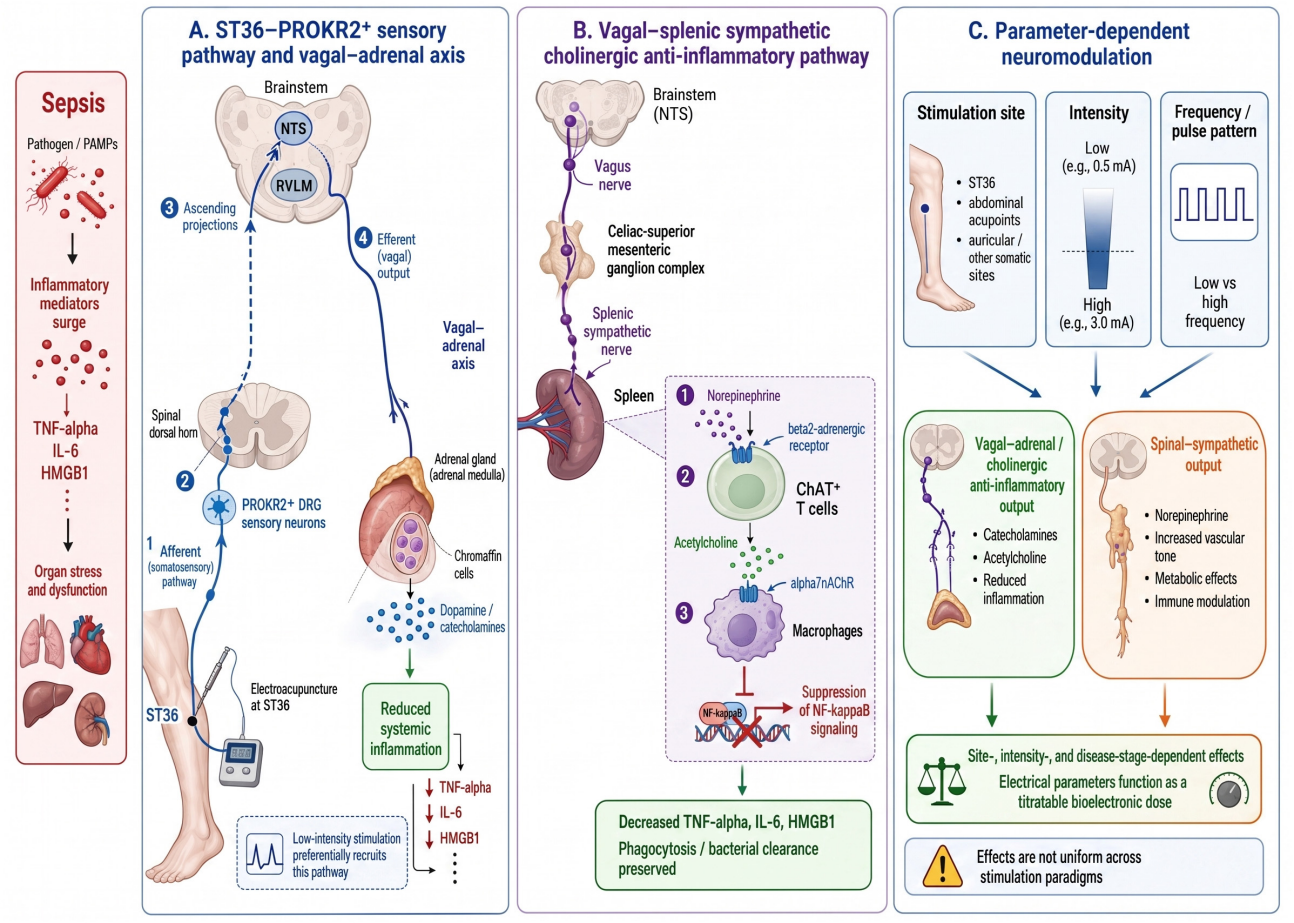
Somatosensory-autonomic circuits engaged by electroacupuncture during sepsis. EA-related neuroimmune signaling should be interpreted as a set of parameter-dependent somatosensory-autonomic circuits rather than a uniform anti-inflammatory intervention. Key pathways include ST36-associated PROKR2+ sensory neurons, vagal-adrenal signaling, and vagal-splenic cholinergic anti-inflammatory communication.

Modern neuroanatomical studies have substantially strengthened the biological plausibility of EA. Experimental work shows that EA effects are somatotopically organized and intensity dependent (9, 29). Low-intensity stimulation at hindlimb ST36 can recruit PROKR2Cre-marked sensory neurons and drive the vagal-adrenal anti-inflammatory axis in mice, whereas high-intensity stimulation may recruit different sympathetic pathways (9). Another conceptual risk is oversimplifying sepsis into hyperinflammation alone. EA may reduce inflammatory mediators in early or hyperinflammatory phenotypes, but patients with profound immunosuppression could theoretically require immune restoration rather than further anti-inflammatory signaling (14). The observation that EA may prevent T-cell lymphopenia in septic mice (25) is therefore intriguing, because it suggests context-dependent immune regulation rather than uniform suppression. Nevertheless, the mechanism is not a single linear pathway. The anti-inflammatory effects of EA involve multiple systems, levels, and targets and should not be reduced to vagal-adrenal activation alone (30). Depending on site, intensity, timing, and disease state, EA may recruit vagal-adrenal, vagal-splenic sympathetic, spinal-sympathetic, hypothalamic-pituitary-adrenal, and local immune-neural pathways. As shown in the left and middle panels of Figure 2, ST36-associated somatosensory afferents may engage vagal-adrenal and vagal-splenic cholinergic outputs; the right panel highlights why stimulation site, intensity, frequency, and disease stage should be treated as dose-defining variables.

### 3.3 ICU confounders that may modify neuroimmune responsiveness

The major translational risk is that neuroimmune circuits demonstrated in controlled models may not behave similarly in septic ICU patients. The PROKR2-ST36-vagal-adrenal axis is primarily established in mouse models and should not be overstated as a proven human ICU mechanism. Septic shock patients frequently receive exogenous catecholamines, particularly norepinephrine as first-line vasopressor therapy (3). This creates a major interpretive problem: exogenous adrenergic support may compete with, mask, or override the endogenous catecholamine signal generated by EA-driven vagal-adrenal activation (9, 10). Vasopressor dose, shock reversibility, adrenergic receptor desensitization, and baseline autonomic failure may therefore determine whether the same electrical stimulus produces measurable neuroimmune output.

Sedation and neuromuscular blockade introduce a second layer of uncertainty. ICU patients commonly receive opioids, propofol, benzodiazepines, dexmedetomidine, or neuromuscular blocking agents according to clinical need, and these therapies alter arousal, nociceptive processing, autonomic tone, ventilator synchrony, and heart-rate variability (HRV)-derived autonomic indices (31, 32). Whether deep sedation or paralysis impairs EA recruitment of PROKR2^+^ somatosensory afferents is unknown. Translational studies should therefore record Richmond Agitation-Sedation Scale scores, analgesic and sedative exposure, neuromuscular blocker use, norepinephrine-equivalent dose, arrhythmia or pacing status, HRV quality metrics, immune phenotype, and organ-function trajectories rather than assuming uniform circuit responsiveness.

Species- and model-specific differences represent another major translational boundary. Most mechanistic pathways discussed above were derived from young, otherwise healthy rodents exposed to LPS or CLP, whereas human sepsis is heterogeneous with respect to age, comorbidity, infection source, pathogen burden, microbiome composition, immune phenotype, timing of treatment, anesthesia exposure, and organ-support intensity (3, 28). Rodent LPS models capture acute endotoxemia rather than the full biology of polymicrobial infection, while CLP more closely resembles peritonitis but still lacks the diagnostic delay, antimicrobial exposure, source-control variability, fluid resuscitation, vasopressors, ventilation, and renal replacement therapy that shape human ICU sepsis. Therefore, rodent EA mechanisms should be interpreted as hypothesis-generating biological signals rather than directly transferable treatment effects.

## 4 Organ-specific mechanisms and clinically meaningful endpoints

**Figure 3:**
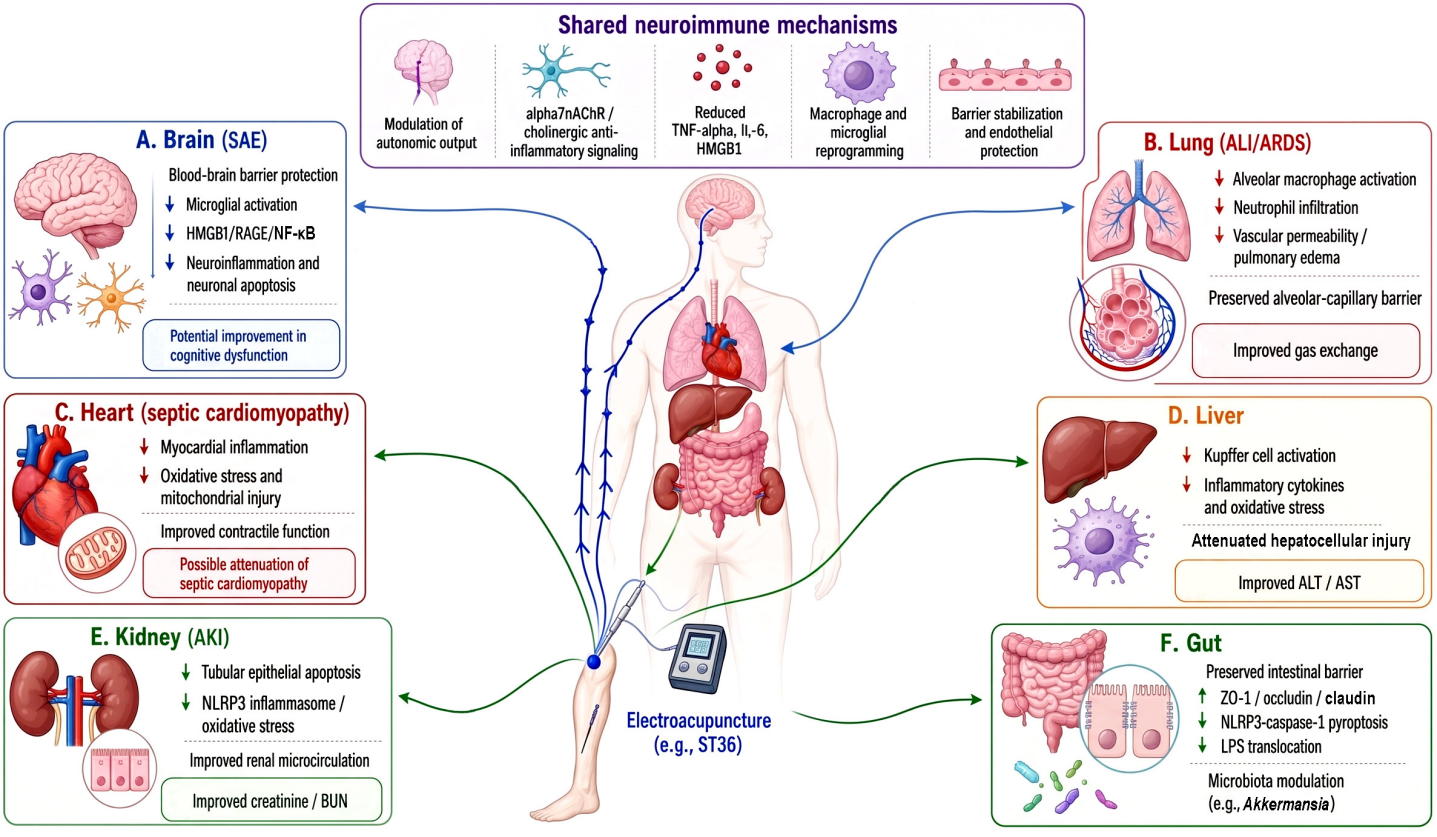
Organ-specific mechanisms of electroacupuncture in sepsis-associated complications. Potential organ effects of EA include gut barrier preservation, reduction of alveolar-capillary injury, suppression of neuroinflammation, attenuation of renal tubular injury, hepatic anti-inflammatory effects, and possible modulation of septic cardiomyopathy. Most non-gastrointestinal organ evidence remains preclinical or exploratory.

Earlier reviews have described broad organ-protective effects of EA in sepsis (11–13). To strengthen clinical relevance, this review reframes organ protection as an endpoint-design problem. The key question is not merely whether EA reduces cytokines in an organ, but which clinically meaningful, organ-specific endpoints should be measured in future trials. Figure 3 organizes the organ-specific discussion by separating shared neuroimmune mechanisms from candidate organ-level endpoints, including gut barrier integrity, alveolar-capillary leakage, neuroinflammation, renal tubular injury, hepatic inflammation, and septic cardiomyopathy.

To avoid implying equivalent readiness across organ systems, the following sections distinguish three levels of evidence: preclinical mechanistic evidence, early clinical signal, and speculative extrapolation. Gastrointestinal dysfunction currently has the most clinically actionable evidence, whereas lung injury/ARDS, sepsis-associated encephalopathy, kidney injury, hepatic injury, septic cardiomyopathy, and ICU-acquired weakness remain predominantly preclinical or exploratory.

### 4.1 Gastrointestinal dysfunction, barrier injury, and gut-derived crosstalk

The intestine is the most coherent organ target for clinical translation. Septic gastrointestinal dysfunction can amplify systemic inflammation through barrier disruption, ileus, dysbiosis, bacterial translocation, intra-abdominal hypertension, and impaired nutrition (33–35). Preclinical studies suggest that EA at ST36 may preserve intestinal mucosal immune and mechanical barriers, maintain tight-junction proteins such as occludin and ZO-1, modulate ghrelin and HMGB1, and reduce epithelial injury (26, 27, 36, 37). More recent work links EA to mitochondrial quality control and HO-1/PINK1-mediated regulation of barrier function (27), and to macrophage polarization in intestinal injury (26).

Clinically, the gastrointestinal literature provides the most actionable endpoints: intra-abdominal pressure, acute gastrointestinal injury grade, abdominal perimeter, bowel sounds, time to enteral feeding tolerance, gastric residual volume, diarrhea or constipation, intestinal fatty acid-binding protein, diamine oxidase, D-lactate, and microbiome or endotoxemia markers (34, 35, 38, 39). These outcomes are closer to the presumed mechanism than mortality and may require smaller trials to detect signal. Therefore, gastrointestinal dysfunction should be considered the preferred first indication for future EA trials in sepsis. The apparent advantage of gastrointestinal outcomes should therefore be interpreted cautiously. It may reflect a biologically plausible gut-autonomic-immune interface: ST36-centered stimulation is closely linked to gastrointestinal motility, vagal signaling, mucosal immune regulation, epithelial tight-junction integrity, barrier permeability, and microbiota-metabolite crosstalk (23, 24, 26, 27, 36, 37). At the same time, it may also reflect evidence-availability bias, because gastrointestinal endpoints such as intra-abdominal pressure, AGI grade, bowel sound recovery, and enteral feeding tolerance have been assessed more frequently than cardiac, renal, neurological, or long-term functional outcomes in existing clinical studies (40–43). Thus, gastrointestinal dysfunction is best viewed as the most clinically developed indication, not as proof that EA is intrinsically more effective for the gut than for other organ systems.

### 4.2 Acute lung injury and ARDS

The lung evidence is promising but mainly preclinical. Early studies showed that EA stimulation at ST36 attenuated LPS-induced acute lung injury in rats (44). Subsequent studies implicated Nrf2/ARE signaling (45), JAK1/STAT3 signaling (46), SIRT1-dependent pathways (47), macrophage polarization (48), and alpha7 nicotinic acetylcholine receptor-mediated anti-inflammatory signaling (49). A translational study reported that EA promoted resolution of inflammation by modulating specialized pro-resolving mediators, particularly lipoxin A4, via vagus nerve and alpha7 nicotinic acetylcholine receptor-dependent mechanisms in LPS-induced acute lung injury, with preliminary human observations in sepsis-related ARDS (50).

Despite these mechanistic advances, ARDS is a high-risk clinical domain. Ventilation strategy, driving pressure, PEEP, prone positioning, fluid balance, neuromuscular blockade, corticosteroid use, and infection source strongly influence outcomes (51, 52). EA trials in lung injury models should therefore avoid relying only on cytokines and should include clinically meaningful ARDS endpoints, including ventilator-free days, oxygenation indices, imaging or lung ultrasound findings when feasible, epithelial/endothelial injury biomarkers, barotrauma, sedative or neuromuscular-blocker exposure, and mortality (53). Registered trials examining EA in sepsis-related ARDS and specialized pro-resolving mediator metabolism should be followed (54), but their results should not be presumed. Accordingly, lung injury/ARDS should currently be considered a preclinical and early-translational domain rather than a clinically ready indication for EA in sepsis.

### 4.3 Sepsis-associated encephalopathy (SAE)

SAE encompasses delirium, impaired consciousness, long-term cognitive impairment, blood-brain barrier dysfunction, microglial activation, oxidative stress, synaptic injury, and neuronal death (55). EA pretreatment or treatment has been reported to attenuate microglial activation, oxidative stress, apoptosis, HMGB1/TLR4 and HMGB1/RAGE signaling, and neuronal pyroptosis in experimental sepsis models (56–60). These studies support a neuroimmune rationale, especially because peripheral neuromodulation may influence central inflammation without direct intracranial intervention.

However, the clinical evidence for SAE remains insufficient. Cognitive outcomes are difficult to measure in sedated, mechanically ventilated patients. Future trials should move beyond inflammatory readouts alone and incorporate clinically meaningful neurologic and cerebral perfusion-related endpoints. Near-infrared spectroscopy may provide dynamic information on regional cerebral oxygenation, whereas transcranial Doppler may estimate cerebral blood-flow velocity and cerebrovascular autoregulation (61, 62). These tools should be combined with delirium assessment, sedation exposure, electroencephalography (EEG) when available, and long-term cognitive outcomes (63), and should be interpreted as adjunctive monitoring modalities rather than definitive evidence of neurologic efficacy. Thus, SAE remains a biologically plausible but clinically unproven target, and future studies should treat neurological outcomes as exploratory until validated in adequately controlled ICU trials (64).

### 4.4 Acute kidney injury and hepatic injury

Kidney and liver evidence is largely derived from endotoxemia models. In LPS-stimulated rabbits, EA ameliorated acute renal injury through PI3K/Akt/Nrf2 signaling and HO-1 induction (65). Earlier rat studies also suggested attenuation of inflammatory responses and acute kidney injury after EA pretreatment (66), and a Wistar sepsis model reported effects on urea and creatinine (67). Hepatic studies indicate that EA at PC6 may attenuate liver injury in endotoxemic rats, and other studies reported effects on hepatic blood flow and lipid peroxidation in septic rats (68).

These data provide mechanistic plausibility but remain far from clinical implementation. Sepsis-associated acute kidney injury is influenced by shock duration, nephrotoxic drugs, venous congestion, rhabdomyolysis, contrast exposure, and renal replacement therapy (69, 70). Hepatic dysfunction is similarly multifactorial. Trials should use KDIGO criteria, renal replacement therapy-free days, creatinine kinetics, urine output, tubular injury biomarkers, bilirubin, coagulation parameters, lactate clearance, and hepatocellular injury markers rather than isolated inflammatory biomarkers (71, 72). Therefore, kidney and liver outcomes should currently be regarded as mechanistic or exploratory domains rather than established clinical indications.

### 4.5 Septic cardiac dysfunction and sepsis-induced myopathy

Septic cardiac dysfunction and ICU-acquired weakness are clinically important because they influence shock persistence, vasopressor exposure, ventilator weaning, rehabilitation, and long-term disability (73, 74). Preclinical studies suggest that EA pretreatment may attenuate sepsis-induced cardiac inflammation and dysfunction through calpain-2/STAT3 signaling (75), and vagus nerve-dependent mechanisms (76). In animal models, this intervention was associated with preserved left ventricular contractility and reduced local cardiac inflammation (76). However, these observations should not be interpreted as proof of clinical cardioprotection. Septic cardiomyopathy is a heterogeneous syndrome involving systolic dysfunction, diastolic dysfunction, right ventricular impairment, altered ventriculo-arterial coupling, myocardial strain abnormalities, and catecholamine-dependent changes in contractility (77, 78).

A key translational issue is the dissociation between macro-hemodynamics and microcirculatory perfusion, often described as hemodynamic incoherence. Septic shock resuscitation can normalize mean arterial pressure and cardiac output while tissue perfusion, endothelial glycocalyx integrity, capillary flow heterogeneity, and mitochondrial oxygen utilization remains impaired. This phenomenon provides a rationale for monitoring microcirculation and tissue perfusion in addition to conventional systemic hemodynamic variables (79, 80). EA-related autonomic modulation could theoretically influence vascular tone and inflammatory endothelial injury, but this signal may be masked or overridden by norepinephrine, fluid loading, inotropes, and vasoplegia. Therefore, future studies should not evaluate cardiac effects solely through inflammatory biomarkers or left ventricular ejection fraction (81, 82). They should incorporate integrated cardiovascular phenotyping, including vasopressor-inotrope dose, lactate clearance, capillary refill time or peripheral perfusion indices, microcirculatory imaging where feasible, bedside echocardiography, and markers of endothelial or mitochondrial injury.

At the cellular level, septic cardiomyopathy involves mitochondrial dysfunction, impaired fatty-acid oxidation, altered glucose utilization, oxidative stress, calcium-handling abnormalities, and inflammatory myocardial depression (74, 83). Future EA studies should therefore test whether neuromodulation modifies myocardial metabolic stress or mitochondrial injury rather than merely reducing circulating cytokines (83). Echocardiographic endpoints should include left ventricular ejection fraction or fractional area change, tissue Doppler indices, E/e′ or other diastolic parameters, right ventricular function, strain when feasible, cardiac output, and vasopressor-inotrope exposure (84). For sepsis-induced myopathy, trials should include Medical Research Council sum score, handgrip strength when feasible, electrophysiology, diaphragm ultrasound, ventilator liberation, mobilization milestones, and long-term functional status (85, 86). Overall, cardiac dysfunction and ICU-acquired weakness remain exploratory targets, and EA-related benefits in these domains require prospective clinical validation before any claim of clinical readiness can be made.

Together, these organ-specific sections show why the evidence matrix in Table 2 and the endpoint hierarchy in Table 3 are needed to separate mechanistic plausibility from clinically actionable trial outcomes.

**Table 3:** Endpoint hierarchy and trial-design priorities for EA studies in sepsis-associated organ dysfunction.

Domain	Preferred primary clinical endpoints	Secondary clinical endpoints	Mechanistic/biomarker endpoints	Key ICU confounders
Gastrointestinal dysfunction	AGI-grade improvement, GI failure-free days, enteral feeding tolerance	IAP, bowel sound recovery, abdominal perimeter, gastric residual volume, diarrhea/constipation, ICU length of stay	I-FABP, DAO, D-lactate, citrulline, tight-junction proteins, microbiome/endotoxemia markers	Sedation, opioids, vasopressors, feeding protocol, abdominal surgery, ileus, fluid balance
ARDS/acute lung injury	Ventilator-free days, oxygenation improvement, mortality in adequately powered trials	PEEP/driving pressure trajectories, lung ultrasound or imaging scores, barotrauma, prone-positioning exposure	Inflammatory mediators, epithelial/endothelial injury markers, SPMs, ferroptosis-related markers	Ventilation strategy, PEEP, driving pressure, prone positioning, fluid balance, NMBA, corticosteroids
Sepsis-associated encephalopathy	Delirium-free/coma-free days, long-term cognitive outcome	CAM-ICU, RASS-adjusted assessments, EEG, NIRS, TCD, sedation-free neurologic assessment	HMGB1/RAGE/TLR4 markers, BBB injury markers, neurofilament, inflammatory cytokines	Sedation, analgesia, hypoxemia, shock, renal/liver failure, baseline cognition
AKI/hepatic injury	KDIGO progression, RRT-free days, AKI-free days; clinically relevant hepatic dysfunction endpoints	Creatinine kinetics, urine output, bilirubin, coagulation parameters, lactate clearance	NGAL, KIM-1, cystatin C, tubular injury markers, hepatocellular injury markers	Shock duration, nephrotoxins, venous congestion, contrast, RRT timing, liver disease
Septic cardiac dysfunction	Shock reversal, vasopressor/inotrope-free days, cardiac dysfunction-free days	LVEF/FAC, diastolic indices, RV function, strain, cardiac output, lactate clearance, peripheral perfusion	Troponin, BNP/NT-proBNP, endothelial injury markers, mitochondrial/metabolic stress markers	Norepinephrine, fluids, inotropes, pre-existing heart disease, arrhythmia, mechanical ventilation
ICU-acquired weakness/myopathy	Ventilator-free days, mobilization milestones, long-term physical function	MRC sum score, handgrip strength, diaphragm ultrasound, electrophysiology	CK, inflammatory markers, neuromuscular injury markers	Sedation, corticosteroids, NMBA, glycemic control, immobilization, rehabilitation intensity
Safety and feasibility	Serious EA-related adverse events, treatment discontinuation, protocol adherence	Bleeding, infection, skin injury, monitoring artifacts, interference with lines/devices	HRV quality, stimulation tolerance, signal quality, adverse-event phenotype	Coagulopathy, thrombocytopenia, ECMO/CRRT, pacemakers/ICDs, edema, staffing and training

## 5 Clinical evidence: signals, uncertainty, and evidence hierarchy

The most comprehensive sepsis-focused meta-analysis included 17 randomized controlled trials and 1,099 patients and reported favorable pooled effects on APACHE II score, inflammatory biomarkers, lactate, intra-abdominal pressure, selected immune indices, and 28-day mortality (40). However, all outcomes were graded as low or very low certainty, and the clinical trial base remains limited by small sample size, usual-care rather than sham comparators, inconsistent blinding, heterogeneous acupoint prescriptions, variable stimulation parameters, and incomplete adverse-event reporting. A key prospective randomized controlled trial in 60 patients with sepsis reported that EA at bilateral ST36 and RN4/CV4 improved APACHE II score and immune indices, including T-cell subsets and monocyte HLA-DR, but did not significantly reduce 28-day mortality (87). In many trials, comparator groups were usual care rather than sham acupuncture, making performance and placebo-related bias difficult to exclude. Mortality reduction should therefore be interpreted as hypothesis-generating rather than clinically established.

Importantly, no current clinical evidence establishes EA as a mortality-reducing intervention in sepsis. Reported mortality signals from meta-analyses should be considered hypothesis-generating because they are derived from small trials with heterogeneous interventions, limited sham control, unclear allocation concealment, inconsistent blinding, and possible publication bias (79, 82). At present, the most defensible clinical interpretation is that EA may produce measurable physiological or biomarker signals, particularly in gastrointestinal dysfunction, but remains unproven for survival, durable organ protection, long-term recovery, or routine ICU implementation (88).

A more systematic appraisal of these clinical studies further limits the strength of inference. Many trials do not clearly report allocation concealment, prespecified primary outcomes, blinded outcome assessment, or complete intention-to-treat analysis. Blinding is particularly challenging because penetrating acupuncture, electrical sensation, practitioner-patient interaction, and acupoint-specific expectations can introduce performance and placebo-related effects. In addition, selective reporting and publication bias cannot be excluded because negative or neutral acupuncture trials may be less likely to be published, and funnel-plot-based assessments are underpowered when the number and size of trials are small. These methodological constraints justify a cautious interpretation of positive clinical signals and support the need for sham-controlled, adequately concealed, prospectively registered, multicenter RCTs with standardized adverse-event reporting (40).

The strongest clinical signal currently concerns sepsis-associated gastrointestinal dysfunction. RCTs of EA at ST36-ST37 reported reductions in inflammatory responses, intra-abdominal pressure, intestinal dysfunction scores, and intestinal barrier-related biomarkers in septic patients (41). A systematic review and meta-analysis focused on septic gastrointestinal dysfunction suggested improvements in intra-abdominal pressure, AGI grade, APACHE II score, abdominal perimeter, and bowel sounds, whereas mortality effects were not robust (42). The EAGISM trial protocol is particularly relevant because it focuses on prevention of acute gastrointestinal injury in mechanically ventilated patients with sepsis and prespecifies ICU-relevant outcomes (43).

**Figure 4:**
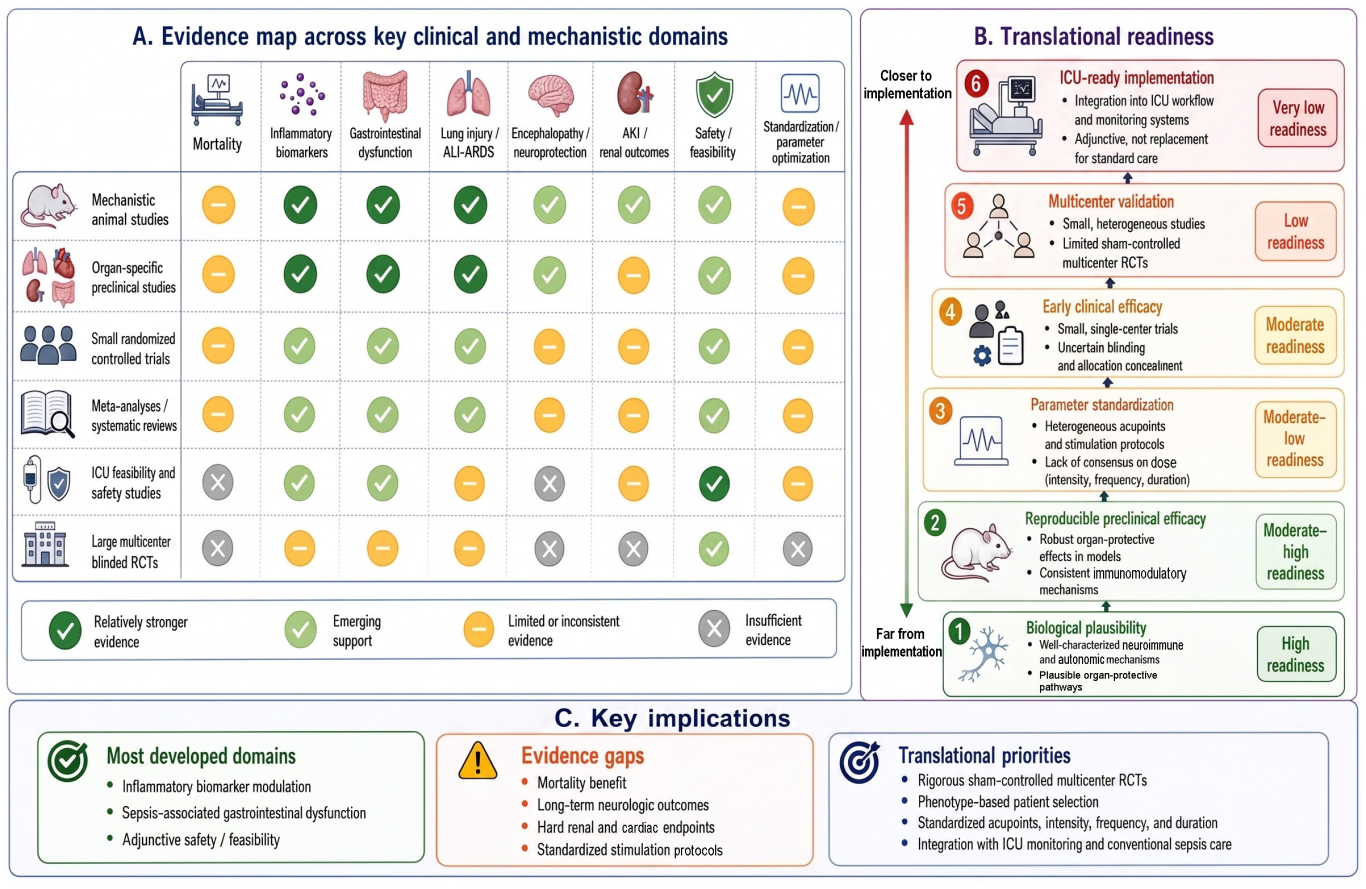
Evidence map and translational readiness of EA-based interventions in sepsis. Current evidence supports biological plausibility and early organ-specific clinical signals, especially gastrointestinal dysfunction, but not confirmed mortality benefit or routine ICU implementation.

This distinction between statistical signal and clinical certainty is central. Existing trials differ in manual acupuncture, EA, transcutaneous electrical acupoint stimulation, acupoint combinations, stimulation frequency, current intensity, session duration, treatment frequency, and timing relative to sepsis onset. These limitations are amplified in sepsis, where source control, antimicrobial timing, fluid balance, vasopressor exposure, nutrition, sedation, mechanical ventilation, renal replacement therapy, and organ-support strategies strongly confound outcomes (3). Figure 4 visually separates biological plausibility from translational readiness, showing that gastrointestinal dysfunction and biomarker modulation are more developed than mortality benefit, cardiac endpoints, renal hard endpoints, and routine ICU implementation.

Because organ protection should be tested with organ-specific clinical endpoints rather than generic inflammatory biomarkers alone, Table 3 translates the current evidence gaps into preferred endpoints, major ICU confounders, and trial-design implications for future EA studies in sepsis. Future trials should prespecify a hierarchy of outcomes. Primary outcomes should be clinically meaningful and organ-specific, whereas inflammatory and barrier biomarkers should be treated as mechanistic or secondary endpoints (88). Without this hierarchy, positive biomarker studies are unlikely to change sepsis guidelines (3).

## 6 ICU implementation: clinical boundaries, contraindications, and safety governance

EA-based interventions in sepsis should be framed strictly as adjunctive neuroimmune modulation, not as an alternative to standard sepsis care. Current sepsis guidelines prioritize early antimicrobials, source control, fluid resuscitation, vasopressors, mechanical ventilation, renal replacement therapy, and organ support when indicated (3). Therefore, EA should never delay or replace these time-sensitive interventions (3); this boundary should be explicitly stated in both clinical protocols and review articles to avoid therapeutic misclassification in a life-threatening infection.

Implementation in the ICU requires predefined safety governance. Although ICU-focused evidence has not shown a clear increase in serious acupuncture-related adverse events, the certainty of safety evidence remains limited because adverse-event definitions and reporting are inconsistent (89). Needle-based interventions should be avoided or deferred in patients with severe thrombocytopenia, disseminated intravascular coagulation, uncontrolled anticoagulation, local skin infection, burns, severe edema at intended acupoints, refractory shock, uncontrolled agitation, or anatomical conflict with extracorporeal membrane oxygenation cannulas, central venous catheters, arterial lines, drains, or surgical wounds (90). WHO safety guidance advises avoiding needling in bleeding and clotting disorders or anticoagulated patients, and a conservative trial threshold of platelet count <50 × 10^∧^9/L is reasonable for invasive needle EA, although no universally accepted acupuncture-specific platelet cutoff exists (91, 92). In patients with platelet counts between 50 and 100 × 10^∧^9/L or unstable coagulation trends, superficial needling, transcutaneous electrical acupoint stimulation, or deferral should be considered.

EA may introduce electrical artifacts into electrophysiological monitoring, including ECG-derived and processed EEG-based systems such as bispectral index; therefore, signal quality should be checked during stimulation and stimulation should be stopped if clinically relevant artifacts occur. Skin should be inspected before and after each session for bleeding, erythema, blistering, pressure injury, edema-related maceration, or local infection. Stimulation should be stopped immediately if new arrhythmia, hemodynamic deterioration, clinically relevant monitoring artifact, unexpected pain, bleeding, skin injury, or interference with life-support devices occurs. In addition, because transcutaneous electrical stimulation has been reported to cause electromagnetic interference with pacemakers and implantable cardioverter-defibrillators, EA should be used cautiously in patients with pacemakers, implantable cardioverter-defibrillators, neurostimulators, or other electrically sensitive devices, particularly when current pathways are close to the thorax or implanted leads (93–95). Device compatibility should be assessed before enrollment, including pacemakers, implantable cardioverter-defibrillators, neurostimulators, and other electrically sensitive devices (96). These risks do not preclude research, but they mandate sterile technique, operator credentialing, standardized documentation of needling depth and electrical parameters, and predefined stopping rules (90).

Practical implementation also depends on feasibility, training, and cost-effectiveness across healthcare systems. Although EA and transcutaneous acupoint stimulation may appear less expensive than many ICU technologies, their true cost-effectiveness cannot be assumed without accounting for practitioner time, credentialing, sterile supplies, device maintenance, monitoring burden, infection-control procedures, workflow disruption, and adverse-event prevention. Multicenter trials should therefore define minimum operator training, competency assessment, standardized stimulation documentation, staff responsibilities, and escalation pathways for safety concerns. In resource-limited settings, simplified non-invasive protocols may be more feasible than needle-based EA, whereas high-resource ICUs may prioritize device compatibility, electronic documentation, and integration with continuous monitoring systems.

Future RCTs should be indication-specific and protocolized. For sepsis-associated gastrointestinal dysfunction, the most clinically developed domain, trials should standardize acupoint prescriptions such as ST36/ST37, define acute gastrointestinal injury grade, and prespecify endpoints including intra-abdominal pressure, feeding tolerance, bowel sound recovery, intestinal barrier biomarkers, SOFA/APACHE II trajectories, ICU length of stay, mechanical ventilation duration, 28-day mortality, and adverse events (39). For ARDS, sepsis-associated encephalopathy, AKI, or ICU-acquired weakness, endpoints should align with organ-specific standards rather than relying only on inflammatory biomarkers, including ventilator-free days and oxygenation indices for ARDS, consensus renal-function criteria for AKI, delirium and cognitive outcomes for SAE, and mobility or muscle-strength outcomes for ICU-acquired weakness (31, 97, 98). CONSORT-STRICTA reporting should be followed to ensure transparent reporting of acupoint rationale, needling details, stimulation frequency, intensity, pulse width, session duration, practitioner background, co-interventions, and sham or usual-care comparators (89). A biomarker-enriched strategy incorporating SOFA/APACHE II, shock status, vasopressor dose, lactate, monocyte HLA-DR, lymphocyte count, cytokine profile, and autonomic tone may help identify patients with preserved neuroimmune responsiveness (15, 16).

### 6.1 Proposed trial framework for sepsis-associated gastrointestinal dysfunction

Because sepsis-associated gastrointestinal dysfunction currently provides the strongest clinical signal and the clearest mechanistic link to autonomic, barrier, mucosal immune, and microbiota-metabolite regulation, it represents the most suitable first target for a phenotype-specific EA trial in sepsis. Table 4 outlines a pragmatic ICU-compatible trial framework that could be adapted to local resources while preserving methodological rigor. EA should be withheld or discontinued if any of these events occur or if urgent ICU procedures or escalating organ support make stimulation unsafe.

**Table 4:** Proposed trial framework for EA in sepsis-associated gastrointestinal dysfunction.

Trial component	Proposed design
Target population	Adult ICU patients with Sepsis-3-defined sepsis or septic shock and early sepsis-associated gastrointestinal dysfunction, preferably AGI grade I-II or mechanically ventilated patients at high risk of AGI.
Key inclusion criteria	ICU admission; expected ICU stay >48–72 h; completed or planned standard source control without delay; hemodynamic status stable enough for non-emergent adjunctive intervention; measurable GI dysfunction or high-risk phenotype.
Key exclusion criteria	Uncontrolled source requiring immediate intervention; refractory shock; severe thrombocytopenia or platelet count <50 × 10^∧^9/L for needle EA; disseminated intravascular coagulation or uncontrolled anticoagulation; local infection, burn, severe edema, or wound at acupoints; uncontrolled arrhythmia; implanted electrical devices at risk; anatomical conflict with lines, drains, or cannulas.
Intervention	Standard sepsis care plus protocolized EA, preferably bilateral ST36/ST37, with predefined frequency, intensity, pulse width, session duration, treatment frequency, operator training, sterile procedures, and treatment window.
Comparator	Standard care plus sham EA or validated sham transcutaneous acupoint stimulation with matched attention, device appearance, patient contact, and session duration when feasible.
Primary endpoint	Change in AGI grade, GI failure-free days, or enteral feeding tolerance within the first 7 days, selected a priori according to trial phase and feasibility.
Secondary endpoints	Intra-abdominal pressure, gastric residual volume, bowel sounds, abdominal distension, diarrhea/constipation, SOFA/APACHE II trajectories, ventilator-free days, ICU length of stay, renal replacement therapy-free days, and 28-day mortality as exploratory outcome unless adequately powered.
Mechanistic endpoints	I-FABP, diamine oxidase, D-lactate, citrulline, tight-junction markers, inflammatory cytokines, monocyte HLA-DR, lymphocyte count, microbiome/endotoxemia markers, and autonomic indices when signal quality is adequate.
Biomarker stratification	Baseline inflammatory phenotype, lymphocyte count, monocyte HLA-DR, cytokine profile, lactate kinetics, shock status, vasopressor dose, AGI grade, and HRV quality if available.
Safety monitoring and stopping rules	Predefined monitoring for bleeding, skin injury, local infection, arrhythmia, hemodynamic deterioration, monitoring artifacts, device interference, severe agitation, urgent procedures, line/drain conflict, or escalating vasopressor requirement.
Reporting standards	CONSORT, STRICTA, prespecified adverse-event definitions, protocol adherence reporting, and intention-to-treat analysis.

## 7 Closed-loop neuromodulation and artificial intelligence: a future perspective

This section is intentionally framed as a future perspective. At present, to our knowledge, there is no direct clinical evidence that AI-guided, adaptive, or closed-loop EA improves outcomes in septic ICU patients. These concepts are discussed only to identify engineering requirements, signal-validation needs, safety constraints, and physician-supervised implementation pathways for future research, not to imply current clinical readiness (99).

A realistic near-term role for AI is decision support rather than autonomous stimulation. AI models could potentially integrate hemodynamic trajectories, vasopressor exposure, ventilator data, lactate kinetics, laboratory trends, infection source, microbiology, bedside ultrasound, and electronic health-record variables to support phenotype recognition, eligibility screening, safety monitoring, and protocol adherence. However, these models would require prospective validation and transparent reporting before being incorporated into EA trials (100–102). Key technical barriers remain unresolved. HRV is not a reliable standalone control signal in many ICU patients because arrhythmias, cardiac pacing, sedation, vasoactive drugs, mechanical ventilation, and signal-processing heterogeneity can distort interpretation (32). Continuous cytokine or sepsis-biomarker biosensing also remains developmental and faces challenges related to sensitivity, calibration drift, biofouling, multiplexing, and regulatory validation (103). Therefore, adaptive dose titration based on real-time immune signals should be treated as speculative.

**Figure 5:**
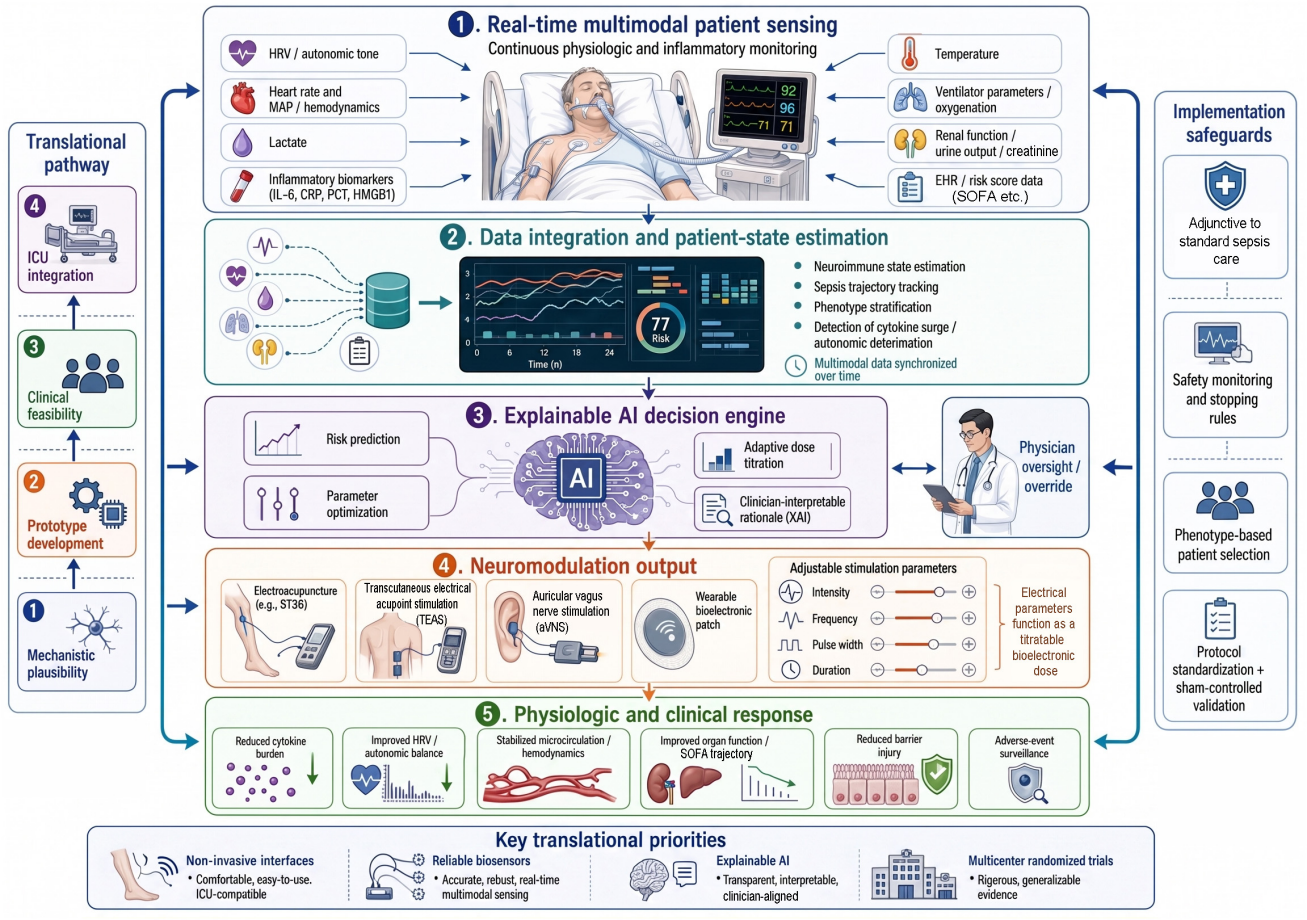
Roadmap for closed-loop electroacupuncture-inspired neuromodulation in the ICU. Future ICU-compatible neuromodulation would require multimodal sensing, explainable AI, clinician oversight, non-invasive or minimally invasive interfaces, and predefined safety constraints. This is a translational roadmap, not current evidence for routine sepsis management.

Any future AI-guided neuromodulation platform should remain physician-in-the-loop. Clinicians must be able to review model inputs, understand the rationale for suggested stimulation changes, override the system, and activate predefined stopping rules. Figure 5 should therefore be read as a roadmap for future research linking wearable interfaces, biosensors, autonomic monitoring, and explainable algorithms, not as evidence that automated EA titration is ready for routine sepsis management.

## 8 Conclusions

EA provides a biologically plausible framework for adjunctive neuroimmune modulation in sepsis by engaging local sensory afferents, somato-autonomic reflexes, vagal-adrenal and cholinergic anti-inflammatory pathways, and downstream immune-cell regulation. The most consistent clinical signal is observed in sepsis-associated gastrointestinal dysfunction, whereas evidence for lung, brain, kidney, liver, cardiac, and neuromuscular complications remains predominantly preclinical or exploratory.

The next phase of research should move beyond broad claims of organ protection toward phenotype-specific, sham-controlled, multicenter trials with standardized stimulation protocols, organ-specific endpoints, biomarker-informed patient selection, and rigorous ICU safety monitoring. EA should be positioned as a biologically plausible but clinically unproven adjunct to guideline-based sepsis care, not as an established treatment for sepsis-induced multiorgan dysfunction. The innovation of future work will depend less on adding another anti-inflammatory pathway and more on making neuroimmune modulation measurable, safe, reproducible, and clinically interpretable in real ICU environments.

## Supplementary material

Supplementary Table S1 Provides additional details on the targeted search strategy and evidence-selection principles used for this critical translational review.
