# Supplementary material for: Electroacupuncture-inspired neuroimmune modulation in sepsis: evidence appraisal and ICU trial-design priorities
Source: Front Med (Lausanne). 2026 Jul 30;13:1891245. doi: 10.3389/fmed.2026.1891245 (PMC13468785; doi:10.3389/fmed.2026.1891245)
Supplement: Supplementary file 1 [file Table_1.docx]

**Supplementary Table S1. Targeted search strategy and evidence-selection principles**

| **Item** | **Description** |
| --- | --- |
| Databases | PubMed/MEDLINE, Embase, Web of Science, Cochrane Library, and ClinicalTrials.gov. |
| Search period | Database inception to May 2026. |
| Sepsis-related terms | sepsis; septic shock; endotoxemia; lipopolysaccharide; LPS; cecal ligation and puncture; CLP; sepsis-associated organ dysfunction. |
| Intervention-related terms | acupuncture; electroacupuncture; transcutaneous electrical acupoint stimulation; TEAS; ST36; Zusanli; ST37; PC6; Neiguan. |
| Mechanistic terms | vagus nerve; inflammatory reflex; cholinergic anti-inflammatory pathway; neuroimmune modulation; vagal-adrenal axis; macrophage; T cell; monocyte HLA-DR; immunosuppression; immune phenotype. |
| Implementation and trial terms | intensive care; ICU; safety; adverse events; contraindication; sham acupuncture; randomized controlled trial; trial design; heart-rate variability; biosensor; artificial intelligence. |
| Eligible evidence | Mechanistic studies, animal sepsis models, human randomized or controlled clinical studies, systematic reviews/meta-analyses, ICU safety literature, reporting guidelines, sepsis guidelines, and clinical trial protocols. |
| Exclusion principles | Duplicated reports; non-sepsis studies without mechanistic or methodological relevance; narrative claims without primary evidence; retracted publications as efficacy evidence. |
| Evidence-selection principles | Priority was given to studies directly addressing sepsis, endotoxemia, CLP, sepsis-associated organ dysfunction, ICU implementation, safety, or clinical trial design. Non-sepsis studies were used only when they clarified general biological or methodological principles and were identified as indirect evidence. |
| Evidence interpretation | Preclinical evidence was treated as biological plausibility; clinical evidence was interpreted according to certainty, methodological limitations, comparator design, and clinical relevance. No quantitative synthesis or formal risk-of-bias scoring was performed. |
| Screening and evidence-selection principles | Records were screened according to relevance to sepsis, endotoxemia, CLP, sepsis-associated organ dysfunction, EA/acupuncture-based interventions, ICU implementation, safety, or trial design. Evidence was prioritized when it directly addressed sepsis-related mechanisms, clinical outcomes, safety, or translational trial design. Non-sepsis studies were retained only when they clarified general biological or methodological principles and were identified as indirect evidence. |
